# Arginine-Derived Cationic Surfactants Containing Phenylalanine and Tryptophan: Evaluation of Antifungal Activity, Biofilm Eradication, Cytotoxicity, and Ecotoxicity

**DOI:** 10.3390/jox15050140

**Published:** 2025-09-03

**Authors:** M. Teresa García, M. Carmen Morán, Ramon Pons, Zakaria Hafidi, Elena Bautista, Sergio Vazquez, Lourdes Pérez

**Affiliations:** 1 Department of Surfactants and Nanobiotechnology, Institute for Advanced Chemistry of Catalonia (IQAC-CSIC), C/Jordi Girona 18-26, 08034 Barcelona, Spain; teresa.garcia@iqac.csic.es (M.T.G.); ramon.pons@iqac.csic.es (R.P.); zakaria.hafidi@iqac.csic.es (Z.H.); elena.bautista@iqac.csic.es (E.B.); sergio.vazquez@iqac.csic.es (S.V.); 2 Secció de Fisiologia, Departament de Bioquímica i Fisiologia, Facultat de Farmàcia i Ciències de l’Alimentació, Universitat de Barcelona, Avda. Joan XXIII 27-31, 08028 Barcelona, Spain; mcmoranb@ub.edu; 3 Institut de Nanociència i Nanotecnologia—IN2UB, Universitat de Barcelona, Avda. Diagonal, 645, 08028 Barcelona, Spain; 4 Doctoral Program in Biotechnology, Facultat de Farmàcia i Ciències de l’Alimentació, Universitat de Barcelona, Avda. Diagonal 643, 08028 Barcelona, Spain

**Keywords:** arginine-phenylalanine and arginine-tryptophan surfactants, surface activity, antifungal activity, biofilm inhibition, cytotoxicity, ecotoxicity

## Abstract

Due to the growing emergence of bacterial and fungal resistance, there is an urgent need for novel antimicrobial compounds. Cationic surfactants are effective antimicrobial agents; however, traditional quaternary ammonium compounds (QACs) are increasingly scrutinized due to their cytotoxicity, poor biodegradability, and harmful effects on aquatic ecosystems. While the antimicrobial efficacy of many new biocides, including QACs, has been extensively studied, comprehensive experimental strategies that simultaneously assess antimicrobial activity, mammalian cell toxicity, and ecotoxicity remain limited. Recent studies have reported that amino-acid-based surfactants containing arginine-phenylalanine and arginine-tryptophan exhibit excellent antibacterial activity and are biodegradable. This work extends their biological characterization to evaluate their potential applications. Specifically, we examined how variations in the head group architecture and hydrophobic moiety influence antifungal and antibiofilm activity. We also assessed how these structural parameters impact cytotoxicity and ecotoxicity. These compounds demonstrated strong activity against a wide range of *Candida* strains. Their hydrophobic character primarily influenced both antifungal efficacy and cytotoxicity. Importantly, these surfactants exhibited potent antimicrobial and antibiofilm effects at non-cytotoxic concentrations. Notably, their aquatic toxicity was significantly lower than that of conventional QACs.

## 1. Introduction

*Candida* species are among the most frequent causes of invasive fungal infections, with Candida albicans being the primary agent responsible for invasive candidiasis. Since the basic cellular structures of fungi are similar to those of human cells, there are relatively few unique molecular targets available for antifungal drugs. Despite the broad negative impact that pathogenic fungi have on public health, they remain a relatively underappreciated contributor to human disease and mortality. Fungal infections affect billions of people each year, with death rates comparable to those of well-known diseases such as tuberculosis and malaria [1]. One of the main reasons for the rising threat of fungal infections is the growing population of immunocompromised individuals [2]. Among fungal pathogens, *Candida* species, especially C. albicans, are the most common source of fungal infections [3]. However, other species such as *Candida glabrata* also cause invasive infections, and more recently, *Candida auris* has emerged as a serious concern in healthcare settings. *C. auris* poses particular challenges due to its ability to colonize human skin, its persistence in hospital environments, and its widespread resistance to treatment [4].

Despite their serious health implications, only a limited number of antifungal drug classes are currently available. In general, the development of new antifungal agents has been notably slow, largely because of the shared eukaryotic structure between fungi and human cells, the difficulty of designing compounds that can penetrate fungal cell walls and membranes, and the limited interest from the pharmaceutical industry due to high research costs and lower financial return [5].

In recent years, numerous *Candida* strains have developed resistance to commercial antifungals. The mechanism of antifungal resistance of *Candida* species has been related to their ability to create biofilms on different surfaces, including numerous medical devices [6]. Therefore, it is urgent to develop new antifungal compounds with potent antifungal and antibiofilm activity, good biocompatibility, and the inability to induce antifungal resistance.

**Figure 1:**
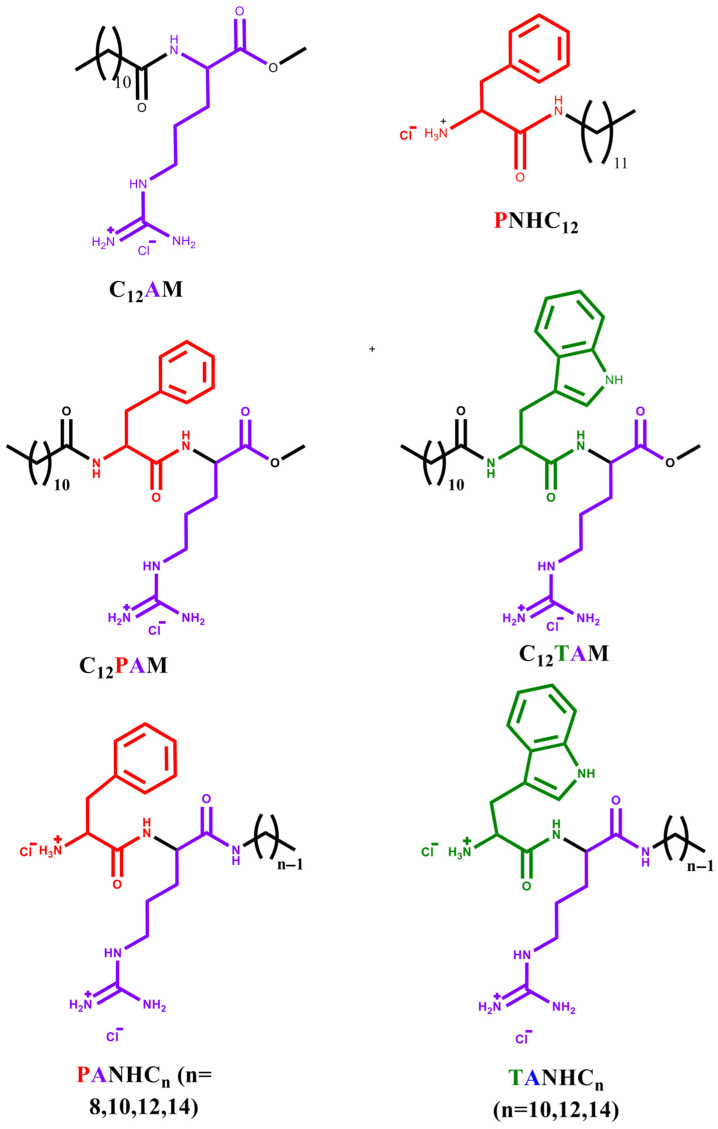
Chemical structures of the amino acid-based surfactants studied in this work.

Cationic quaternary ammonium surfactants (QACs) have good antibiofilm and antifungal activity; however, these surfactants usually show toxicity against mammalian cells, and after use, the residual products are discharged to sewage treatment plants or directly to the water surface. Considering that many cationic surfactants have high aquatic toxicity [7], the environmental impact is of great concern. Therefore, the main challenge in the development of new cationic antimicrobial surfactants is to achieve an effective balance between antimicrobial efficacy, toxicity toward mammalian cells, and environmental sustainability. Although extensive research has been undertaken to assess the potency of many biocides, including QACs [8,9,10,11], their cytotoxicity and ecotoxicity have been little assessed. There is also a lack of existing experimental approaches that combine examinations of both antimicrobial potency and toxicity against mammalian cells and aquatic organisms. Recently, our research group reported the synthesis and properties of phenylalanine and tryptophan-based cationic surfactants [12]. The products were synthesized from renewable starting products, including amino acids, fatty acids, and fatty amines, and their chemical structure (head group, hydrophobic group, and alkyl chain position) was systematically modified (Figure 1). The critical micelle concentration of these surfactants mainly depends on the hydrophobic/hydrophilic balance. These compounds were easily biodegraded under aerobic conditions, with the extent of degradation depending on the amino acid in the hydrophobic moiety and the position of the hydrophobic group [12]. It was found that these surfactants exhibited very good activity against a wide range of Gram-positive and Gram-negative bacteria, including some problematic pathogens such as MRSA and *L. monocytogenes*. Typically, the mode of action of surfactants against bacteria involves disrupting cell membranes, resulting in the leakage of intracellular components. Considering all these biological properties, it can be assessed that these cationic surfactants exhibit strong potential as novel antibacterial agents with prospective biomedical applications.

However, the antifungal and antibiofilm activities of these compounds, as well as their toxicity toward mammalian cells and aquatic organisms, have not yet been reported. Therefore, this study aims to deepen the understanding of the relationship between the chemical structure of cationic amino acid-based surfactants and their antifungal and antibiofilm efficacy against several clinically relevant *Candida* strains. In addition, we evaluated their cytotoxicity using two mammalian cell lines—fibroblasts and keratinocytes—and assessed their ecotoxicological impact. Furthermore, we investigated their surface activity and self-aggregation behavior to explore correlations between their molecular structure and physicochemical properties.

## 2. Methods

### 2.1. Synthesis

The synthetic procedure used for preparing these compounds and their structural characterization are widely explained in [12]. The retention time of these surfactants was measured by HPLC (Merck-Hitachi D-2500, Merck, Darmstadt, Germany) using a LiChrosphere 100 CN column 5 μm, 250 × 4 mm. The solvent system used was aqueous phase, trifluoroacetic acid 0.1% (*v*/*v*) in H_2_O, and organic phase, trifluoroacetic acid 0.095% (*v*/*v*) in H2O/ACN (1:4). UV-detector at λ = 215 nm and flow rate of 1 mL/min.

### 2.2. Surface Tension Measurements

Surface tension was measured using a custom-built pendant drop tensiometer, as described in detail in reference [13]. Briefly, a droplet of surfactant solution is formed at the tip of a straight-cut Teflon tube. The image of the droplet is captured using a webcam, and its shape is analyzed by fitting the droplet profile to the Laplace–Young equation [14], To minimize evaporation, all measurements were conducted in a saturated humid atmosphere. The use of this technique is especially well-suited for cationic surfactants because it helps to mitigate complications related to surface adsorption [15]. Critical micelle concentration values (cmc) were determined by finding the intersection of the linear fits before and after the break in the curves.

The logP values, reflecting the lipophilicity of the compounds, were determined using the Molinspiration Cheminformatics web-based property prediction tool (https://www.molinspiration.com/cgi/properties (accessed on 14 May 2025)). The tool employs a fragment-based approach to predict molecular properties, including logP, by calculating the contributions of predefined substructural fragments. This approach is widely applied in medicinal and computational chemistry due to its high efficiency and accuracy.

### 2.3. Antimicrobial Activity

*(a)*  *Microorganisms and Culture Conditions*

The antimicrobial activity of these compounds was assessed against selected yeast and bacterial strains: *Candida albicans* ATCC 90028 (CA9), *Candida rugosa* ATCC 10571 (CR), *Candida glabrata* ATCC 66032 (CG), *Candida parapsilosis* ATCC 22019 (CP), *Candida tropicalis* ATCC 7349 (CT), *Candida auris* ATCC 21092 (CA), and *Cyberlindnera jadinii* ATCC 60459. Frozen yeast stocks were plated on Sabouraud agar (SBA) and incubated at 30 °C for 24 h. Three to four colonies from each strain were then suspended in RPMI 1640 (Roswell Park Memorial Institute medium, Sigma- Aldrich, Merck, Darmstadt, Germany) to prepare bacterial dispersions of ~1.5 × 10^6^ CFU/mL.

*(b)*  *Minimum Inhibitory Concentration (MIC)*

A broth microdilution assay was used to calculate MIC values. Twofold serially diluted compounds in RPMI 1640 medium (from 256 to 2 μg/mL), were dispensed (100 μL per well) into a 96-well plate. Then, 100 μL of a yeast starter culture was added to each well to reach a final inoculum of 7.5 × 10^5^ CFU/mL. Wells containing only nutrient broth without the tested surfactant served as growth controls. Microbial growth was assessed by turbidity, which reflects increases in biomass and cell count. MIC was defined as the lowest concentration at which no visible growth occurred after 24 h of incubation at 30 °C. To confirm the obtained MICs, 20 μL of 0.015% (*w*/*v*) resazurin was dispensed to each well and incubated for 3 h at 30 °C. Metabolic activity was evidenced by a color change from blue to pink, thereby confirming the MIC value.

### 2.4. Antibiofilm Activity

*Candida* strains were cultured in SBA at 30 °C for 24 h. Yeast cells were then suspended in RPMI 1640 broth to a final concentration of 1.5 × 10^7^ CFU/mL. For mature biofilm formation, 200 μL of these suspensions was transferred to each well of a 48-well microplate and incubated at 30 °C for 24 h. After incubation, the plate was washed with phosphate-buffered saline (PBS) to remove non-adherent cells. Then, surfactant solutions at various concentrations (200 μL), prepared in RPMI 1640, were added to the wells containing the mature biofilms. Plates were incubated again at 30 °C for 24 h. Following this treatment, the RPMI medium was removed, and wells were washed twice with PBS. To assess biofilm viability, 200 μL of MTT solution (3-(4,5-dimethylthiazol-2-yl)-2,5-diphenyltetrazolium bromide) were added to each well, and plates were incubated for 4 h. After incubation, the MTT solution was removed, and 200 μL of dimethyl sulfoxide (DMSO) were added to solubilize the formazan crystals. Absorbance at 540 nm (A_540_) was then measured using a microplate reader. Controls without the amino acid-based surfactants were included. All experiments were made in quadruplicate, and the results were averaged to ensure accuracy and reproducibility.

### 2.5. Hemolytic Activity Assay

Fresh rabbit blood was obtained from the Animal Facility of the CID-CSIC (Research and Development Center, Spanish National Research Council, Barcelona, Spain). Sample collection was conducted in strict compliance with the bioethical guidelines established under Spanish legislation. Erythrocytes were isolated by centrifugation (3000 rpm/10 min). The supernatant was discarded, and the erythrocytes were washed three times with PBS (phosphate-buffered saline, pH 7.4) and then resuspended in PBS at a concentration of 8 × 10^9^ cells/mL. Subsequently, varying volumes of a concentrated surfactant solution were added to Eppendorf tubes containing 25 μL of erythrocyte suspension. PBS was then added to bring the total volume to 1 mL. The samples were gently shaken for 10 min at room temperature and centrifuged at 10,000 rpm for 5 min. Hemolysis was evaluated by measuring the absorbance of the supernatant at 540 nm and comparing it to that of a 100% hemolysis control (erythrocytes treated with distilled water). The HC_50_, defined as the surfactant concentration that induces 50% hemolysis, was determined from the concentration–response curves:

$$\%\;hemolysis=\;\frac{Abs_{test\;compound}}{Abs_{Basal\;\;haemolytic\;activity}\;}\times 100$$

### 2.6. Cytotoxicity Assays

Immortal human keratinocyte cells (HaCaT) and human fibroblast cells (3T3) were obtained from Celltec, University of Barcelona. Dulbecco’s Modified Eagle Medium (DMEM) (4.5 g/L glucose) supplemented with 10% (*v*/*v*) FBS, 2 mM L-glutamine, 100 U/mL of penicillin, and 100 μg/mL of streptomycin was used to grow the cells in 75 cm^2^ culture flasks at 37 °C with a continuous supply of CO_2_ at a 5% level. Once cells reached about 80% confluence, they were trypsinized with trypsin-EDTA.

Cell Viability Assays

Cells were first grown into the central 60 wells of a 96-well plate under 5% CO_2_ at 37 °C for 24 h. The DMEM was removed, and cells were exposed to different concentrations of each amphiphile diluted in DMEM supplemented with 5% FBS for 24 h. Finally, two different assays for quantification were used.

(a) Neutral Red Uptake (NRU Assay)

The NRU assay is based on the accumulation of neutral red dye within the lysosomes of viable, intact cells. Once the cells were incubated with the surfactants, the DMEM was removed, and the NRU dye solution (50 μg/mL dissolved in the medium without FBS and phenol red) was added to each well, and the reaction was allowed for 2 h. The cells were washed with PBS, and 100 μL of an aqueous solution containing 50% absolute ethanol and 1% acetic acid was then added to each well to extract the incorporated dye. The microplates were then stirred for 5 min at 25 °C to facilitate dissolution. Finally, the absorbance of the resulting solutions was measured at λ = 550 nm using a Bio-Rad 550 microplate reader (Hercules, CA, USA).

(b) MTT Assay

This method relies on the ability of living cells to reduce the yellow MTT reagent into insoluble purple formazan crystals. After the cells were incubated with the surfactants’ solutions, the medium was eliminated, and 100 μL of MTT in PBS (5 mg/mL) diluted 1:10 in culture medium (without phenol red and FBS) was added to each well and co-incubated for 3 h. Then, the medium was discarded, and DMSO was added into each well to solubilize the formazan crystals. Finally, the absorbance of the resulting solutions was measured at 550 nm using a microplate reader (Bio-Rad 550 Hercules, CA, USA).

### 2.7. Ecotoxicity Assays

*Acute Toxicity Assessment Using Daphnia magna*

Aquatic toxicity evaluation was performed using *Daphnia magna* following established protocols [16]. This bioassay method employs laboratory-cultured freshwater microcrustaceans to evaluate surfactant toxicity in aquatic environments. Test organisms were utilized during their initial 24 h life stage to ensure consistency. The endpoint measurement focused on mobility impairment, specifically the loss of swimming capability after gentle stimulation lasting 15 s. Testing conditions included controlled water chemistry parameters: pH maintained at 8.0, calcium-to-magnesium ratio of 4:1, and total hardness of 250 mg/L as CaCO_3_. All experiments were conducted under dark conditions at 20 °C. The experimental design incorporated ten test concentrations for each surfactant compound, distributed in geometric progression. Each concentration level included 20 *Daphnia* specimens distributed across four replicate groups of five organisms each. Mobility assessment occurred at 48 h post-exposure, with immobilization percentages plotted against concentration using probability–logarithmic scaling. Statistical analysis employed probit methodology via SPSS software (IBM SPSS Statistics 27.0.1) to determine the median inhibitory concentration (IC50)—the concentration causing 50% immobilization—along with 95% confidence intervals for each tested compound.

*Vibrio fischeri Luminescence Reduction Test*

Ecotoxicological assessment of cationic surfactants was conducted using the *Vibrio fischeri* luminescent bioassay [17]. This method relies on the measurement of decreased bioluminescent output from marine bacteria following contact with potentially toxic substances. Regression analysis was used to determine the concentration of the compound that caused a 50% reduction (EC_50_) in the amount of light emitted by the bacteria. The stated toxicity statistics given are based on the bacteria being exposed to the ionic liquid solution for 15 min at 15 °C.

## 3. Results and Discussion

There is a growing interest in the development of new antifungal agents to address the increasing problem of fungal resistance. The amino acid-based surfactants derived from arginine, tryptophan, and phenylalanine described in this study can be considered “green” compounds, as they align with several principles of green chemistry: they are synthesized from renewable resources and are readily biodegradable [12]. While our previous work focused on their antimicrobial properties and mechanism of action against bacteria, their antifungal and antibiofilm activities remained unexplored. The current study aims to bridge this gap by investigating how the chemical structure of cationic amino acid-based surfactants influences their antifungal and antibiofilm effectiveness against several clinically relevant *Candida* strains. We have studied surfactants in which we systematically varied key structural parameters: the number and type of amino acids in the polar head group, the length of the alkyl chain, and the position of the hydrophobic moiety (either at the α-amino or carboxylic group of the amino acid). Based on these structural modifications, we classified the compounds into two distinct sets of surfactants, as illustrated in Figure 1:

(a) Six monocatenary surfactants in which, while keeping a constant C12 alkyl chain, we systematically varied the polar head in terms of the amino acid type, the number of cationic charges, and the attachment position of the alkyl chain: **C_12_AM** (N^α^-dodecyl arginine methyl ester hydrochloride), with a C_12_ hydrophobic chain connected to the α-amine group of arginine and a positive charge on the protonated guanidine group; **PNHC_12_** (phenylalanine dodecyl amide hydrochloride), with the cationic charge on the protonated amine group of the amino acid, and the C_12_ chain is linked to the carboxylic group of the phenylalanine; **C_12_PAM** (N^α^-dodecyl phenylalanine-arginine methyl ester hydrochloride**)** and **C_12_TAM** (N^α^-dodecyl-tryptophan-arginine methyl ester hydrochlorid**e),** with two aromatic amino acids on the hydrophilic moiety (Phe/Arg or Trp/Arg), one positive charge on the protonated guanidine group, and the C_12_ chain connected through an amide linkage to the α-amino group of the aromatic amino acid; **PANHC_12_** (phenylalanine-arginine dodecyl amide dihydrochloride) and **TANHC_12_** (tryptophan-arginine octyl amide dihydrochloride) compounds that also contain two amino acids on the polar head (Phe/Arg or Trp/Arg) but with two cationic charges (one on the protonated amine group of the aromatic amino acid and the other in the protonated guanidine group), and the C_12_ alkyl chain is linked to the carboxylic group of the arginine (Figure 1).
(b) Seven monocatenary surfactants in which, keeping constant the polar head with two amino (Phe/Arg or Trp/Arg) and two cationic charges, we varied the alkyl chain from C8 to C14 for the Phe/Arg and C10 to C14 for the Trp/Arg homologs: PANHCn (phenylalanine-arginine alkyl amide) and TANHCn, (tryptophan-arginine alkyl amide).

The synthesis and structural characterization of all these new surfactants are explained in detail in [12].

### 3.1. Surface Tension

Surface tension measurements as a function of surfactant concentration are a basic characterization technique to know the surface activity of amphiphile molecules. Increasing the concentration of surfactant reduces the surface tension of water due to adsorption. Usually, this reduction levels off at a concentration often identified as the critical micelle concentration, although this identification has long been discussed [15].

**Figure 2:**
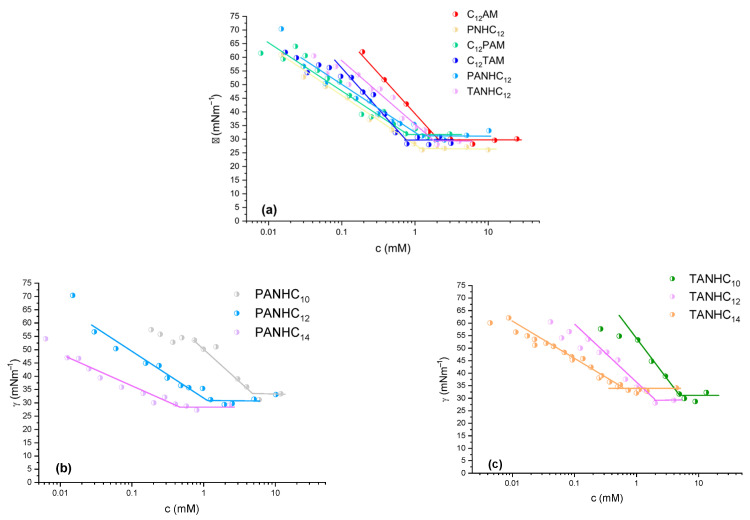
Surface tension against surfactant concentration for the amino acid-based surfactants. (**a**) C_12_ alkyl surfactants, (**b**) PANHCn homologs and (**c**) TANHCn homologs.

For the homologous series with a C_12_ alkyl chain (Figure 2a), the cmc appears to be mainly influenced by the hydrophobicity of the amino acids in the polar head group. The highest cmc value was observed for C_12_AM, which contains only an arginine with a cationic charge (Table 1). Introducing an additional hydrophobic amino acid into the hydrophilic moiety leads to a reduction in cmc, particularly in the cases of C_12_PAM and C_12_TAM, surfactants featuring an aromatic amino acid in the polar head and a single positive charge. This trend can be partly attributed to the predominantly ionic nature of these surfactants, where the hydrophobic substituents on the polar head may behave more like additional hydrophobic domains than true polar head modifiers [18]. Figure 2b,c illustrate the influence of hydrophobic chain length, revealing the expected trend of approximately halving the cmc with each additional methylene group in the alkyl chain. In general, the cmc obtained by surface tension measurements is similar to that determined by conductivity, although some discrepancies were observed, a common occurrence in surfactants involving acid-base equilibria, because bulk micellization behavior can differ from interfacial properties.

Table 1 also shows the HPLC retention time (Rt) and the logP for these compounds. HPLC retention time is often used to compare relative hydrophobicity within a series of structurally related compounds. Hydrophobic molecules interact more strongly with the nonpolar stationary phase and elute more slowly; hence, they show longer retention times. As expected, this parameter correlates with the obtained cmc values. LogP is the logarithm of the partition coefficient (P) of a compound between octanol and water. It quantifies how hydrophobic (lipophilic) or hydrophilic a molecule is. The logP values also correlate with the cmc, surfactants with higher logP values have lower cmcs. The logP values fall within the range 0–3, indicating that these compounds are neither highly hydrophilic nor highly lipophilic. Regarding the surface tension at the critical micelle concentration (γ_cmc_), only minor variations were observed across the series, ranging from 26 to 33 mN·m^−1^, with no clear trend associated with changes in the polar head group or alkyl chain length.

### 3.2. Antifungal Activity

Their antifungal activity of these compounds was evaluated by determining their MIC against seven representative ATCC *Candida* strains.

**Figure 3:**
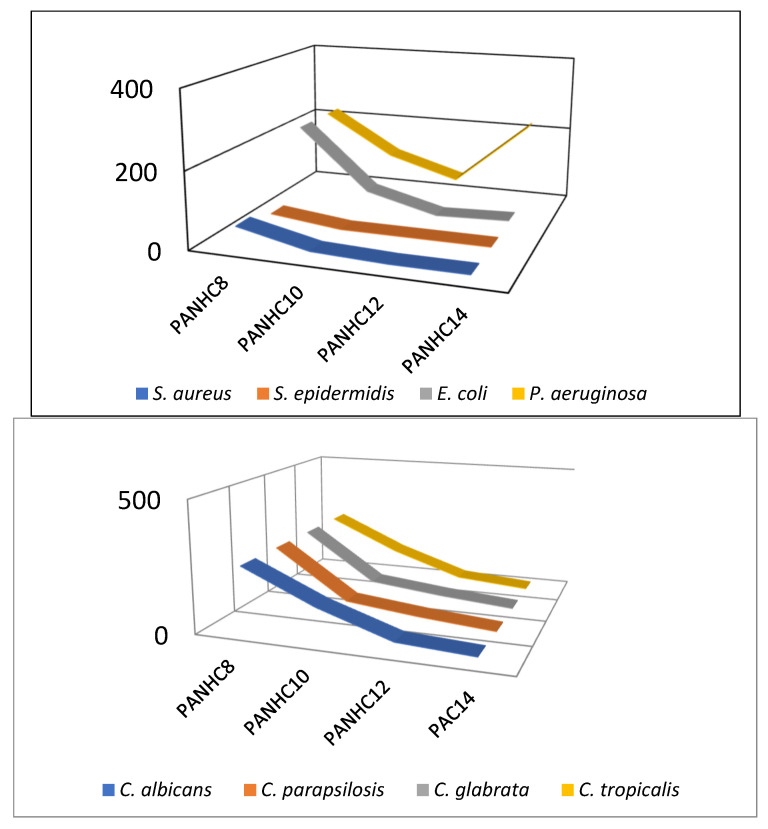
Effect of the alkyl chain length on the antibacterial and antifungal activity of the PANHCn. Antibacterial activity from reference [12].

Table 2, Figure 3, and Figure S1 show the MIC values obtained for compounds with C_12_ alkyl chains. These surfactants exhibited good activity against all *Candida* strains tested. The strong antifungal effectiveness of these surfactants can be attributed to their chemical structure and specific mode of action. These surfactants possess one or two positive charges, one on the protonated arginine guanidine group that, given its high pKa value, hardly undergoes deprotonation. Furthermore, both the amide bond and the guanidine group in their structures can form hydrogen bonds with components of biological membranes, enhancing their membrane affinity. Based on their chemical structure, these compounds are expected to act through a combination of electrostatic and hydrophobic interactions with fungal membranes. These interactions facilitate the intercalation of the cationic surfactants into the membrane, leading to its disruption. Furthermore, the compounds may penetrate the cell and interfere with essential intracellular functions [19,20]. In this regard, we have recently observed that the mode of action of two of the cationic surfactants studied in this work, C_12_TAM and C_12_PAM, against fluconazole-resistant *Candida* strains involves alteration in the cell membrane permeability and mitochondrial damage [21]. Antimicrobials targeting the cell membrane can contribute to reducing antimicrobial resistance, as it is challenging for microorganisms to develop resistance against agents that disrupt this essential structural barrier in bacteria and fungi [22].

Among these C_12_ analogs, C_12_PAM and C_12_TAM exhibited slightly superior antifungal activity, with MIC values ranging from 4 to 32 μg/mL, compared to MIC values between 8 and 128 μg/mL observed for C_12_AM, PNHC_12_, PANHC_12_, and TANHC_12_. Table 2 also contains the MIC values of benzalkonium chloride, one of the most potent antiseptics of the mono QAC type, widely used in domestic, cosmetic, and pharmaceutical applications. This surfactant also contains an aromatic group in the polar head. Notice that the MIC values of BAC are similar to those observed for the C_12_PAM and C_12_TAM. Regarding the effect of the aromatic group on the antimicrobial activity of cationic surfactants, different results can be found in the literature. For instance, benzalkonium bromide demonstrated greater activity than its counterpart, dodecyl trimethyl ammonium. In contrast, *Ghost* et al. found that for cationic amphiphiles with long hydrophobic chains, the addition of an aromatic group reduced their antimicrobial efficacy [23].

One of the highlights of this set of compounds, especially the C_12_PAM and C_12_TAM, is their good activity toward the opportunistic human pathogen *C. auris*. Currently, *Candida auris* has emerged as a significant concern in healthcare settings due to its resistance to treatment and its rapid development of resistance to antifungal agents. Analysis of numerous *Candida auris* isolates revealed that 80% were resistant to fluconazole, 23% to amphotericin B, and 7% to micafungin. Additionally, 24% of the isolates exhibited resistance to at least two classes of antifungal agents, while approximately 1% were resistant to all three major antifungal classes [24]. It was also observed that, among these surfactant families, the two N^α^-dodecyl-diamino acid derivatives demonstrated the highest activity against certain problematic bacteria, such as *Listeria monocytogenes* and *Acinetobacter baumannii* [12]. Notably, these two surfactants also exhibited the highest levels of biodegradability, which may also be attributed to the specific positioning of their alkyl chains.

Keeping the polar group constant, the alkyl chain has been varied in the PANHCn and TANHCn. The alkyl chain length, which determines the hydrophobic/hydrophilic balance of surfactants, is another structural parameter that strongly influences their antimicrobial potency. Table 3 presents the MIC values of tryptophan- and phenylalanine-based homologs with C8–C12 alkyl chains. The data obtained for these two series demonstrated that their inhibitory effect on the growth of *Candida* strains is strongly associated with variation in chain length. For both types of surfactants, the antifungal efficiency increases as the alkyl chain increases. The PANHC_8_, the homolog with the shortest alkyl chain, exhibited activity only against three of the strains tested (*C. tropicalis*, *C. parapsilosis* and *C. glabrata*) at the highest concentration tested, while the C_10_ derivatives, especially the PANHC_10,_ had moderate activity against all microorganisms. Then, while all C_12_ amphiphiles displayed antimicrobial potency, their MICs varied from 32 to 64 μg/mL, the C_14_ homologs showed the strongest broad-spectral potency against all *Candida* strains, with MIC values around 4–16 μg/mL. This is a common trend observed across many cationic surfactant homologs. Generally, within a homologous series, increasing the length of the alkyl chain enhances antibacterial and antifungal activity up to an optimal point, beyond which further elongation leads to a decline in efficacy [25]. The optimum alkyl chain length depends on the chemical structure of surfactants; for the monocatenary cationic surfactants, usually the C_12_-C_14_ derivatives show the best antimicrobial activity [26,27] while for gemini surfactants, often higher activity was observed with shorter chain length [28,29]. The best activity of the PANHCn and TANHCn homologs for bacteria was observed for the C_12_ derivatives, while for fungi, the C_14_ homologs were more active (Figure 3). This behavior was already observed for monocatenary surfactants from arginine [30] and can be ascribed to the different composition of the fungal membranes. The inner cytoplasmic membrane of *Staphylococcus aureus* consists of approximately 58 mol% negatively charged phosphatidylglycerol (PG) and 42 mol% cardiolipin, both anionic phospholipids. In contrast, the inner membrane of *Candida albicans* contains only about 30% anionic compounds. Additionally, C. albicans is surrounded by a hydrophobic, glucan-rich cell wall, which provides an extra barrier to external agents [31]. Due to this different membrane composition, the optimum alkyl chain for these surfactants was observed for the most hydrophobic compound, which probably improves the hydrophobic interaction with the fungal membranes.

The MIC values of these compounds are consistently lower than their cmc, indicating that these surfactants interact with all *Candida* strains primarily in their monomeric form.

The relationship between cmc and MIC appears to be influenced by the correlation between alkyl chain length and both parameters. The relationship between cmc (critical micelle concentration) and MIC (minimum inhibitory concentration) is influenced by the alkyl chain length of the surfactants. As the length of the alkyl chain increases, log cmc decreases linearly (see Table 1). This linear trend follows Kleven’s equation, which gives log cmc = 3.019–0.23n for the PANHCn series, and log cmc = 3.11–0.27n for the TANHCn series, where n is the number of carbon atoms in the alkyl chain. However, the relationship between MIC and alkyl chain length is not as straightforward. MIC values drop sharply up to the C_12_ chain length, but beyond that (from C_12_ to C_14_), they either level off or decrease more slowly. As a result, there is not a linear relationship between cmc and antimicrobial activity. These results suggest that the hydrophobicity of the molecule affects cmc and MIC differently, which explains why cmc does not have a linear correlation with MIC.

Interestingly, these amino-acid based surfactants were effective against a wide range of problematic *Candida* strains, pathogens that nowadays cause problematic *Candida* infections. Changes on the polar head slightly affect the antifungal activity, while changes on the hydrophobic alkyl chains strongly affect their efficiency. The antimicrobial activity against Gram-positive and Gram-negative bacteria behaves similarly. The antifungal activity of the C_12_ and C_14_ homologs is similar to that exhibited for arginine-based rhamnolipids [32] and other potent monoquats such as C_14_TAB and DDAB [31,33,34].

The surfactants investigated in this study may be classified as ultrashort lipopeptides, compounds composed of a fatty acid moiety linked to short cationic peptides (3–8 amino acids), that are regarded as promising candidates for combating bacterial and fungal pathogens. Currently, efforts are being made to reduce the relatively high production costs of these compounds by further shortening the peptide sequence while preserving both antimicrobial efficacy and biocompatibility. The shortest peptide chains in the polar head usually contain a minimum of three or four amino acid residues, frequently combinations of arginine or lysine with other amino acids [35,36,37,38].

In this study, very good antimicrobial amphiphiles with just two amino acid residues have been prepared. By shortening the peptide part to two residues, the molecules with C_12_ and C_14_ alkyl chains exhibit high antimicrobial efficacy toward a wide range of pathogens, including Gram-positive bacteria, Gram-negative bacteria, and fungi. Their minimum inhibitory concentrations (MICs) are comparable to those reported for the most potent short lipopeptides and peptoid analogs bearing two amino acid residues in their polar heads [39]. Notably, these amphiphiles were synthesized via a green, cost-efficient method, underscoring their potential as sustainable alternatives in antimicrobial development.

### 3.3. Antibiofilm Activity

**Figure 4:**
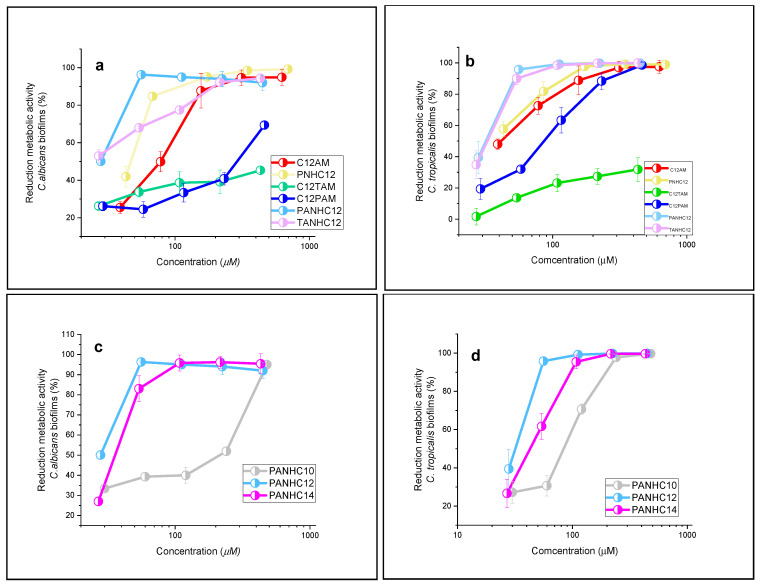

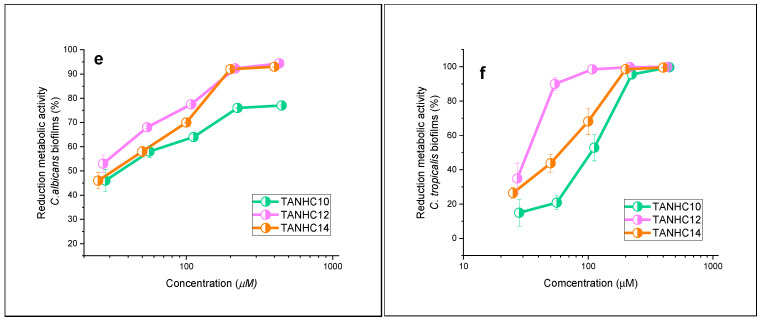
Quantification of reduction in viable cells in *C. albicans* (**a**,**c**,**e**) and *C. Tropicalis* (**b**,**d**,**f**) biofilms by the MTT method. Error bars are generated from six replicates.

In this section, we have tested the ability of these cationic surfactants to eradicate mature fungal biofilms of *C. albicans* and *C. tropicalis*. Preformed biofilms of these two strains on 48-well plates were exposed to various concentrations of the synthesized surfactants, and the biofilm was not removed after the treatment was quantified using the MTT method (3-[4,5-dimethylthiazol-2-yl]-2,5 diphenyl tetrazolium bromide) (Figure 4). This method detects viable and metabolically active cells within the biofilm by measuring the reduction in MTT to formazan by active mitochondrial enzymes. We have used two *Candida* strains, *C. albicans,* which is the most frequently occurring fungal species associated with biofilms, and *C. tropicalis*, a non-*C. albicans* species that exhibits innate resistance to most azole-based drugs and has emerged as an important cause of candidiasis [40].

Our results indicate that the antibiofilm activity of the surfactants is concentration dependent. Notably, most of these compounds reduced the biofilm viability at relatively low concentrations. The concentrations required to achieve complete biofilm inactivation were higher than their corresponding MIC values. During the intermediate phases of biofilm formation, *C. albicans* changes its morphology from yeast to hyphae, and *C. tropicalis* forms pseudohyphae [41]. Moreover, these biofilms contain a polysaccharide matrix that restricts the diffusion of antifungals inside the biofilm. So, the resistance of fungal pathogens in biofilms usually increases significantly compared to the planktonic form. For example, the MBEC (Minimum Biofilm Eradication Concentration) of multicationic quaternary ammonium compounds against *C. albicans* is about 7 times their MIC values [42], and the MIC values of CTAB, ciprofloxacin, chlorhexidine, and tetracycline against *S. aureus* SH1000 were 2, 4, 1, and 0.5 µg/mL, respectively, but the MBEC of all these compounds was >256 µg/mL [43].

The efficiency to eradicate mature biofilms is influenced by the number and type of amino acids on the polar head and by the length of the hydrophobic alkyl chain. Figure 4a,b show the antibiofilm activity of the C_12_ homologs with different polar heads. For the same hydrophobic alkyl chain, the activity against these two *Candida* strains changed significantly by changing the number of amino acids on the polar head as well as the position of the alkyl chain. The best activity against both microorganisms was obtained for compounds with only one amino acid on the hydrophilic group (C_12_AM and PNHC_12_) and surfactants with two amino acids and the alkyl chain linked to the carboxylic acid (PANHC_12_ and TANHC_12_). However, the C_12_PAM and C_12_TAM, surfactants with the hydrophobic chain connected to the amine group of the aromatic amino acid, displayed lower antibiofilm activity. Both compounds (C_12_PAM and C_12_TAM) reduced less than 65% of the metabolic activity of the *C. albicans* at the highest concentration tested. These differences can be ascribed to the polar head volume and the number of cationic charges on the molecule. The main reason for the *C. albicans* biofilm resistance is the extracellular matrix. This ECM is composed of different biological molecules such as carbohydrates, glucose, β-1,3-glucan, etc. Moreover, some negatively charged molecules such as eDNA, eRNA, and uronic acid are also present in the ECM [44]. On one hand, compounds with less bulky polar head groups, such as C_12_AM and PNHC_12_, may exhibit enhanced diffusion through the biofilm matrix, facilitating their antibiofilm action. On the other hand, the negatively charged components of the extracellular polymeric matrix could promote stronger electrostatic interactions with surfactants bearing two cationic charges, such as PANHC_12_ and TANHC_12_, potentially increasing their antibiofilm efficacy.

Figure 4c,d show the effect of alkyl chain length on the antibiofilm activity of the PANHCn against the two *Candida* species. There is a clear trend toward an increase in the antibiofilm activity with the enlargement of their alkyl chain. Thereby, the C_10_ homolog removed the total viability of these biofilms only at the highest concentration tested (≈400 μM) while the C_12_ and C_14_ homologs disrupted all biofilms at a concentration as low as ≈50 μM. A similar trend was observed for the tryptophan-based derivatives (Figure 4e,f). This behavior is commonly reported for cationic surfactants, where the optimal alkyl chain length for biofilm disruption varies depending on the specific chemical structure of each surfactant series. For compounds containing two arginines and one alkyl chain, the best efficiency removing the *candida* biofilms was observed for the C_14_ homolog [37]. For cholinium-based ionic liquids and surfactants with two cationic charges on the polar head, the best antibiofilm activity was found for the C_16_ derivative [45]. For gemini surfactants from arginine, the best antibiofilm activity was found for the C_10_ analog [46]. For gemini quaternary ammonium salt, compounds with C_16_ alkyl chains showed the best antibiofilm activity [47], and for tris-Quaternary ammonium, compounds with C_10_ alkyl chains showed the lowest MBEC values against *C. albicans* [48].

These results indicate that cationic surfactants with the arginine amino acid on the polar head have good antifungal activity and remove *Candida* biofilms at low and effective concentrations. Notably, the efficiency of our best surfactants is comparable to that of cetylpyridinium chloride [42]. Similar ability to eradicate established *C. tropicalis* and *C. albicans* biofilms was observed for N^α^-benzoyl-arginine-alkyl-amide [49] and cationic amphiphiles with two arginines on the polar head [37].

### 3.4. Cytotoxicity

Evaluating cytotoxicity is crucial for determining the potential applications of antibacterial and antifungal surfactants. However, although extensive research has been undertaken to assess the potency of many biocides, including QACs, their cytotoxicity has been little studied. In this work, the interaction of the prepared amino acid-based surfactants with mammalian cells has been evaluated using erythrocytes and two human cell lines (3T3 and HaCaT).

**Figure 5:**
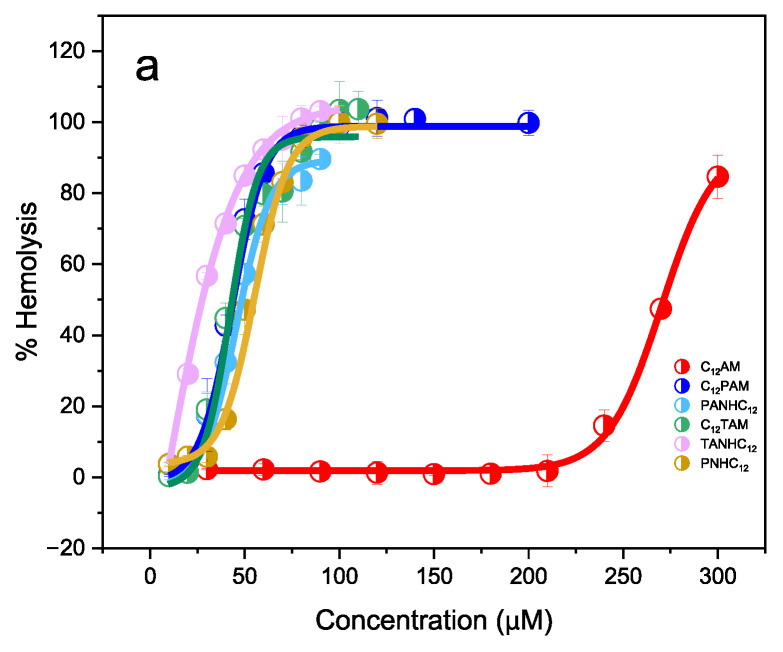

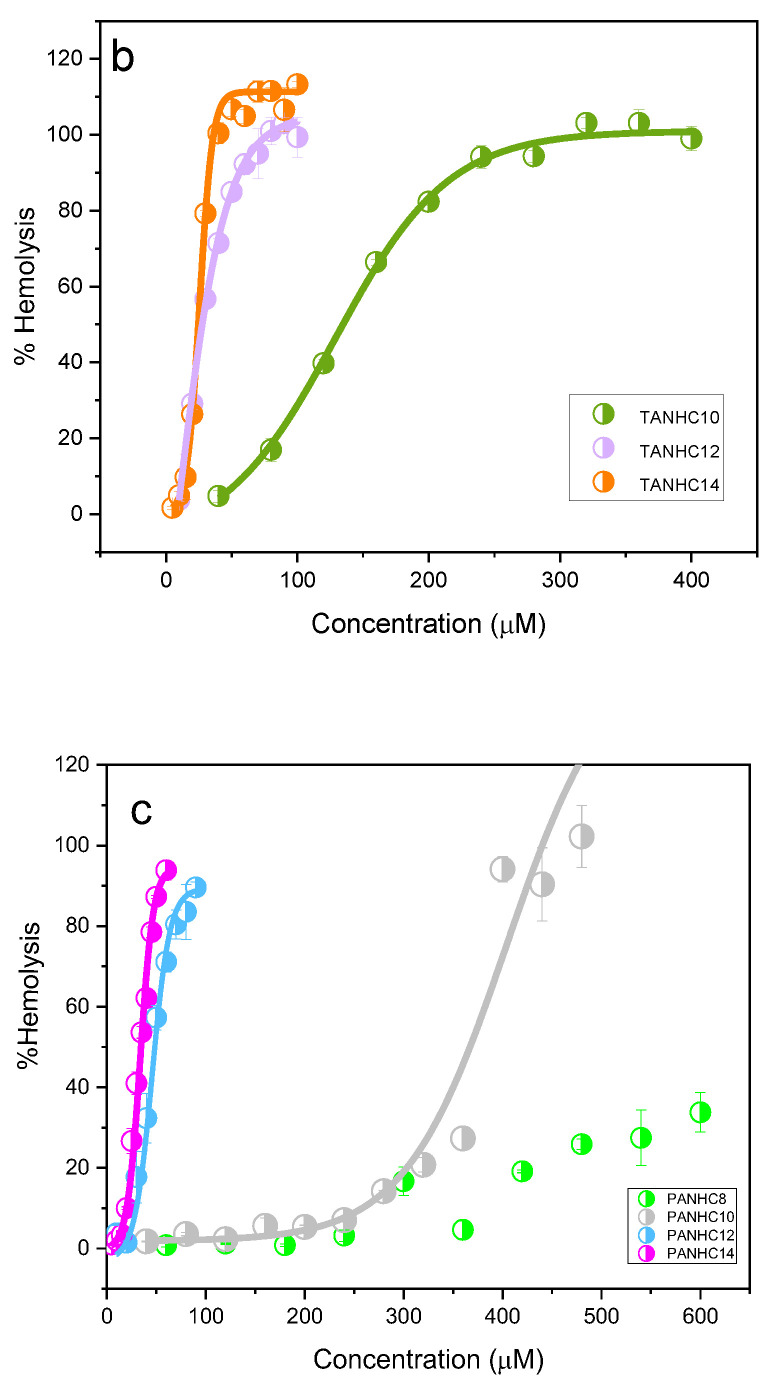
Curves of hemolysis (%) against surfactant concentration for the C_12_ homologs (**a**), TANHCn (**b**), and PANHCn (**c**).

Erythrocytes, extremely fragile cells that lack internal organelles, are one of the most commonly used cells to measure surfactant cytotoxicity. The hemolytic activity of these surfactants was determined in erythrocytes from rabbit blood samples. We sought out to systematically study the effect of the aromatic amino acid on the polar head (keeping the alkyl chain constant), as well as the effect of the alkyl chain length, keeping constant the polar head. The evaluation of the HC_50_, the concentration at which 50% of the red blood cells are lysed, was quantified from plots of percentage of hemolysis as a function of surfactant concentration (Figure 5). Table 4 summarizes the obtained HC_50_ values.

When the alkyl chain length is kept constant (C12 homologs) and the polar head is varied—whether by the number of amino acids, the type of aromatic residue, or the position of the alkyl chain—distinct HC_50_ values are observed (Figure 5a). Among these compounds, C_12_AM displayed by far the lowest hemolytic activity. This reduced toxicity can be attributed to its lower hydrophobicity, as indicated by its retention time (Rt) and critical micelle concentration (cmc) (Table 1). The PHNC_12_, a surfactant with just one phenylalanine on the polar head, had a higher hydrophobic character and lower HC_50_ value than the C_12_AM.

For the C_12_ derivatives, incorporating an aromatic amino acid—either phenylalanine or tryptophan—into the polar head led to an increase in hemolytic activity. Notably, C_12_PAM and C_12_TAM can be regarded as analogs of C_12_AM, differing only by the insertion of an aromatic residue between the alkyl chain and the arginine. This modification significantly affects cytotoxicity, as the HC_50_ value of C_12_AM is approximately five times higher than that of C_12_TAM and C_12_PAM. This performance can be attributed to the higher hydrophobic character of the C_12_TAM and C_12_PAM; notice that these compounds exhibited lower cmc values and higher retention times (Table 1). Regarding the type of amino acids, only a slight decrease in the hemolytic character was observed for surfactants containing arginine and tryptophan. Our results indicate that the alkyl chain position did not affect the hemolytic activity; the HC_50_ values of surfactants with the alkyl chain linked to the amine group of the amino acid (C_12_PAM, C_12_TAM) were similar to that of the surfactants with the alkyl chain connected to the carboxylic acid of the amino acid (PANHC_12_, TANHC_12_). In contrast, this structural change significantly impacted the biodegradation of these compounds. While C_12_AM, C_12_PAM, and C_12_TAM achieved over 60% biodegradation, as determined by the CO_2_ Headspace test, PNHC_12_, PANHC_12_, and TANHC_12_ did not reach this pass level [12]. The HC_50_ value corresponding to BAC has also been included in Table 4. The data show that the hemolytic activity of this quaternary ammonium salt is slightly lower than that of the cationic surfactants containing two amino acids on the polar head, but significantly higher than that of C12AM. This behavior can be attributed to the hydrophobic nature of the molecules. The BAC used in this study is a mixture of C12 (70%) and C14 (30%) alkyl chain homologs, with a critical micelle concentration (cmc) of 5.2 mM, as determined by conductivity measurements. This indicates that the hydrophobic character of BAC is comparable to that of C12AM and substantially greater than that of the C12 surfactants containing either arginine/phenylalanine or arginine/tryptophan.

If the polar head is kept constant, and the alkyl chain is varied, series PANHCn and TANHCn, important changes in the hemolytic activity were observed (Figure 5b,c). Compounds with short alkyl chains, C_8_ and C_10_, exhibited high HC_50_ values (Table 4). On moving from C_10_ to C_12,_ an important decrease in the HC_50_ values was obtained; the HC_50_ value of the PANHC_10_ was 404 μg/mL while that of the PANHC_12_ was 45.7 μg/mL. After this, on moving to the C_14_ analogs, the hemolytic activity continues increasing but with lower intensity. Usually, the longer the aliphatic chain is, the greater the hemolytic activity [25]. But it has also been reported that, for some surfactant families, the cytotoxicity also presents the “cutoff” effect on their alkyl chain length, similar to the antimicrobial activity. In this regard, Sikora et al. found that the hemolytic character of tryptophan-based surfactants and glycosylated lipopeptides containing two arginines in the polar head [50] decreased by increasing their hydrophobic content.

The cytotoxic effects of these cationic surfactants were also evaluated using two different cell lines. The use of in vitro cytotoxicity tests is increasing daily, driven by the rise in human exposure to chemicals, as these tests are less costly and time-consuming than in vivo methods. The purpose of in vitro cytotoxicity tests is to detect the biocompatibility of these new surfactants. In this work, the cellular response upon incubation with the different surfactant series was evaluated in two representative skin cell lines: Swiss mouse fibroblasts (3T3 cell line) and human-derived keratinocytes (HaCaT cell line). The in vitro cytotoxicity was determined using the MTT as a measure of mitochondrial activity and the Neutral Red Uptake (NRU) related to lysosomal integrity, which are the most commonly used in vitro cytotoxicity endpoint methods. The cellular response at different concentrations (5, 25, 50, and 100 µM) was evaluated after 24 h of incubation.

**Figure 6:**
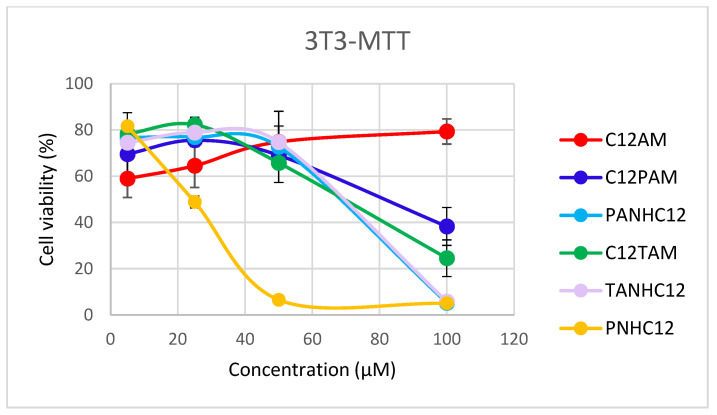

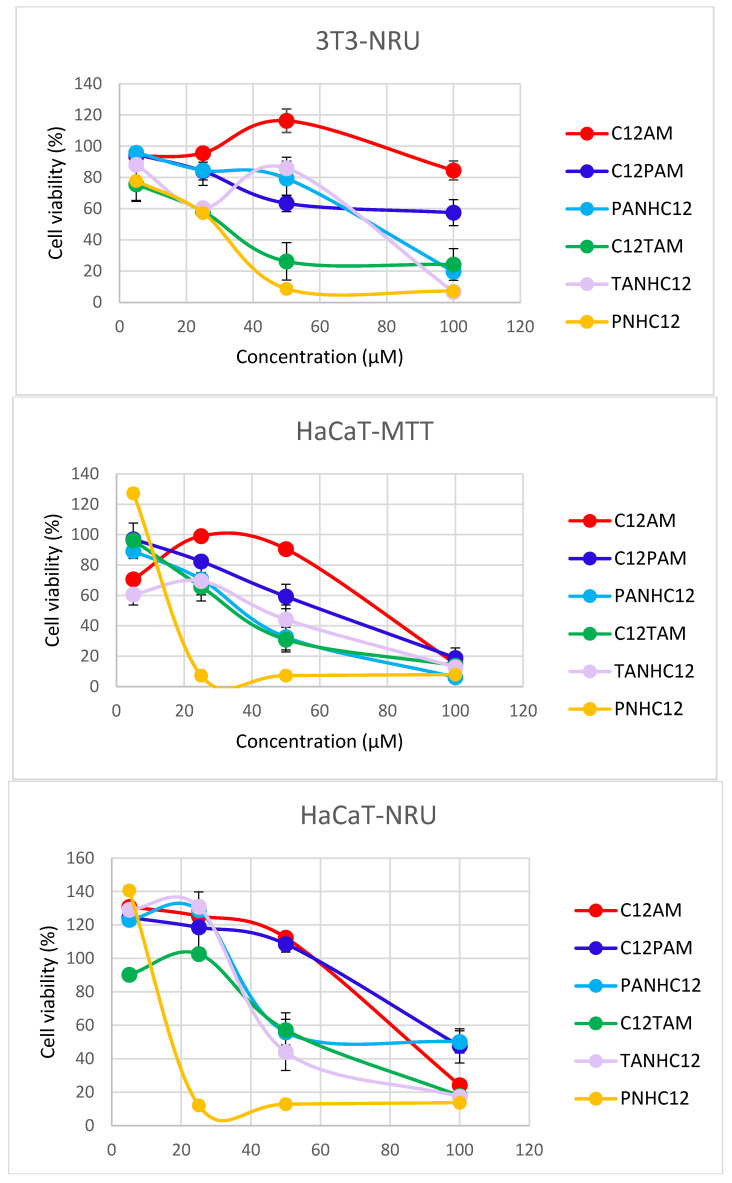
Concentration–response curves from 24 h exposure of C_12_ homologs to the 3T3 cells and HaCaT cells.

Figure 6 shows the cytotoxic effects corresponding to the C_12_ homologs. All these surfactants exhibit a clear dose–response relationship. The effects of the C_12_ derivatives on 3T3 cell viability responses determined by MTT assay report high viability for C_12_AM (>85%) across all concentrations up to 100 µg/mL. C_12_PAM exhibited mild toxicity, with a slight decrease in viability starting around 50 µg/mL, reaching approximately 60% at 100 µg/mL. In the case of PANHC_12_ derivative, cell response showed an initial mild toxicity observed at 50 µg/mL (viability around 70%), dropping to 5% at 100 µg/mL. Similar results were observed for the TANHC_12_ and C_12_TAM; their viability decreases steadily between 50 and 100 µg/mL. These results reflect cumulative mitochondrial damage in this cell line. Different results were observed for the PNHC_12_; this compound promoted severe mitochondrial dysfunction even at low concentrations, viability dropped to nearly 0% at 50 µg/mL. These results indicate that the IC_50_ of these compounds is between 50 and 100 µg/mL, except for PNHC_12_, in which the IC_50_ is lower than 50 µg/mL. Considering the viability obtained at 100 µg/mL and 50 µg/mL, the cytotoxicity of these compounds follows the following trend: PNHC12 > TANHC12 > TANHC12 > C12TAM > C12PAM > LAM. For the HaCaT cells, MTT assay results revealed that these cells are more sensitive to these amino acid-based surfactants. At a concentration of 100 µg/mL, cell viability dropped below 80% for all compounds. At 50 µg/mL, the trend in cytotoxicity was similar to that observed for the 3T3 cell line. The most cytotoxic compound was PHNC_12_, which reduced cell viability to 0%, whereas C_12_AM was the least toxic, maintaining approximately 90% viability.

The 3T3 cell responses by the NRU method provided a similar trend to MTT. The C_12_AM maintained 100% viability across the full concentration range. C_12_PAM confirms biocompatibility, retaining lysosomal integrity with viabilities ranging between 60 and 100% at the entire concentration range. Both TANHC_12_ and PANHC_12_ showed moderate toxicity around 50 µg/mL, whereas PNHC_12_ showed dramatic toxicity that began at 5 µg/mL, confirming extreme lysosomal disruption, consistent with MTT results. The cellular responses against HaCaT using the NRU are similar to those observed with the MTT endpoint. The viability results indicated that the least cytotoxic compounds were the C_12_AM and C_12_PAM, whereas the PNHC12 exhibited the highest toxicity.

These results demonstrated high biocompatibility for C_12_AM with no significant toxicity up to 100 µg/mL. C_12_PAM, C_12_TAM, PANHC_12_, and TANHC_12_ exhibited moderate cytotoxicity, with mitochondrial damage preceding lysosomal effects. PNHC_12_ was highly toxic to both 3T3 and HaCaT cells, affecting mitochondria and lysosomes at very low concentrations. HaCaT cells were more sensitive overall, especially in NRU assays, indicating tissue-specific vulnerabilities relevant for skin exposure risk. Concerning the endpoint methods. MTT assays typically showed earlier and more pronounced toxicity, making them slightly more sensitive for detecting early cell stress.

Similar toxicity against human fibroblast 1BR.3.G cells was obtained for the N-Carbobenzyloxy–arginine-NHC_12_, a cationic surfactant also containing a benzyl group and an arginine on the hydrophilic moiety [51]. However, the IC_50_ values of the C_12_ analogs studied in this work were higher than those reported by Joondan et al. for a C_12_ derivative consisting of one polar head of phenylalanine-proline with a C_12_ alkyl chain and one cationic charge on the trimethylated amine group of the phenylalanine [52].

**Figure 7:**
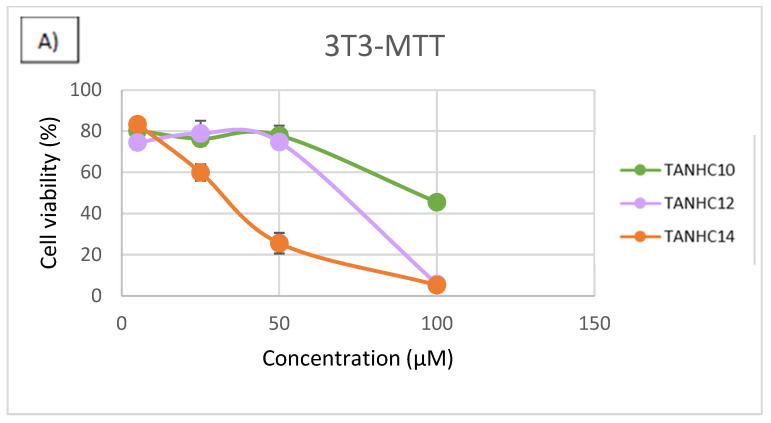

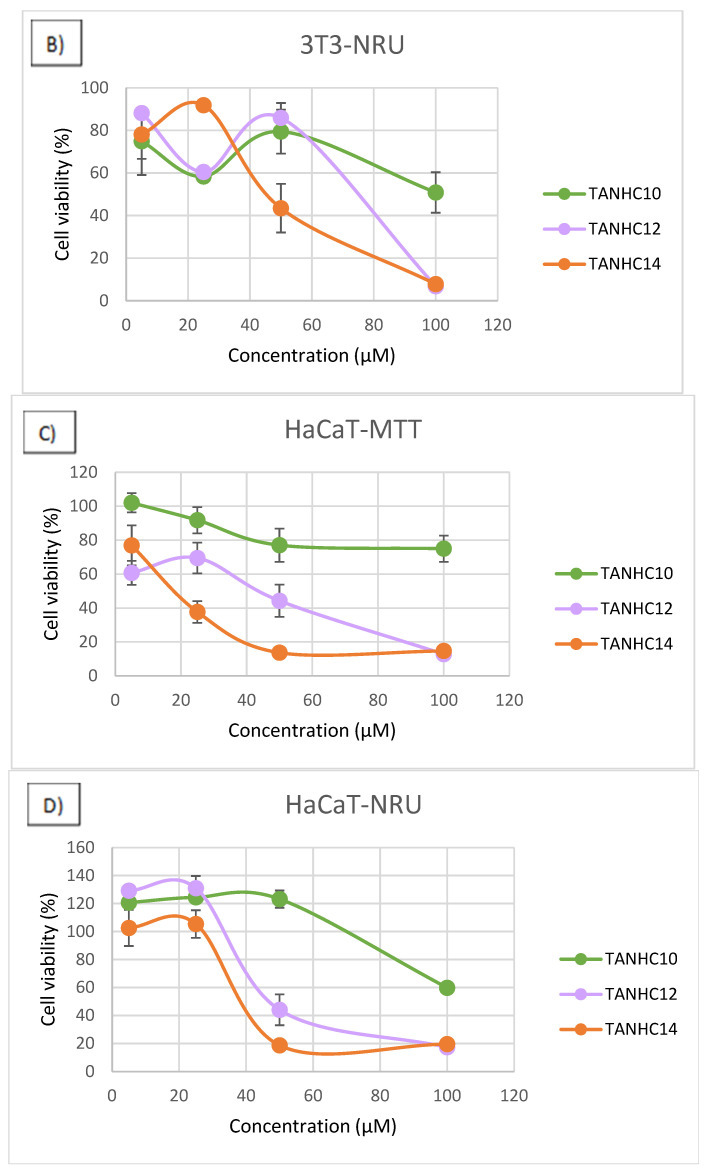
Concentration–response curves from 24 h exposure of TANHCn homologs to the 3T3 cells (**A**,**B**) and HaCaT cells (**C**,**D**).

To establish the effect of the alkyl chain on the toxicity of these surfactants against mammalian cells, the cytotoxicity of the TANHCn series was determined (Figure 7). A clear influence of the hydrophobic chain length was found for the two cell lines tested. In the case of 3T3 fibroblasts, the shorter derivative (TANHC_10_) shows the highest viability across all concentrations. By increasing the chain length (TANHC_12_), the viability showed a gradual decrease. The fourteen derivative (TANHC_14_) was found to be the most cytotoxic, reducing viability significantly after 25 µM. In the case of HaCaT keratinocytes, a similar trend was observed; the TANHC_10_ maintained higher viability (approximately 80%) across the concentration range, but TANHC_12_ and TANHC_14_ promoted more toxicity. By the NRU assay, the viability of 3T3 increased slightly before decreasing (possibly a hormetic effect). Again, TANHC_10_ was least toxic, with moderate decreases in viability at higher doses. The viability of HaCaT cells by the NRU assay initially increased, suggesting some cell proliferation or lysosomal stimulation, especially for the shorter compounds (TANHC_10_ and TANHC_12_).

These results demonstrated that the hydrophobic content of the molecule is a critical factor in cell viability. On one hand, for the C_12_ derivatives, the C_12_AM, the most hydrophilic surfactant, exhibits the least cytotoxic character. On the other hand, increasing the alkyl tail (from 10 to 14) leads to stronger cytotoxic effects in both cell lines. The TANHC_10_ was the least cytotoxic compound in both cell lines and across both assays, indicating its potential biocompatibility, whereas TANHC_14_ was consistently the most cytotoxic. Previous results obtained by our group also confirm that the hydrophobicity seriously affects the cytotoxicity; it was found that the HC_50_ values of surfactants containing two polar amino acids and two cationic charges (lysine-lysine or lysine-arginine) were considerably higher than those obtained for gemini surfactants with higher hydrophobic content [53].

Comparing the obtained results with literature data, we can conclude that these new amino acid-based surfactants have less cytotoxic effects against human cell lines than commonly used QAS such as cetylpyridinium chloride (C_16_TAB), and didecyldimethyl ammonium chloride (DDAB) [54]. Similar toxicity has been reported for pyridinium-based surfactants using NIH3T3 cells; in this case, the C_15_ homolog of this molecule showed similar cytotoxicity to the surfactants studied in this work, more than 50% viability up to a concentration of 100 μg/mL [55]. Quaternary ammonium leucine-based surfactants with C_12_ alkyl chains and an aromatic benzyl group in the polar head also exhibited similar HC_50_ values, but the IC_50_ against Caco cells were significantly lower (0.016 mM and 0.013 mM for the C_12_ and C_14_ analogs). The cytotoxicity of these leucine-based surfactants exhibited a similar trend to that observed for the surfactants presented here. By changing the alkyl chain from C_10_ to C_12,_ the HC_50_ and EC_50_ values decrease significantly (from 160 μM to 16 μM); then, from C_12_ to C_14_, only a slight decrease in cytotoxicity was observed [56].

In general, the toxicity of the surfactants studied here is similar to that reported in the literature for several short lipoamino acids. For example, Dawgul et al. observed that the C_16_ single-chain homolog of cationic lipopeptides containing two or three amino acids had a IC_50_ < 10 μg/mL whereas the IC_50_ of the C_10_ double-chain derivative was 42.1 μg/mL [36]. Neubauer et al. also synthesized short cationic lipopeptides, and the cytotoxicity of compounds with similar hydrophobicity to the surfactants described here, showed comparable HC_50_ values [37]. A similar trend was observed by Chao Zhong et al.; these authors prepared ultrashort lipopeptides with three amino acids on the polar head (combinations of arginine and tryptophan) and different alkyl chains. They observed that the hemolytic activity of these compounds increased significantly with the increasing hydrophobicity in a fatty acid chain length-dependent manner [57].

Notably, the HC_50_ of some of these compounds and the IC_50_ against the two cell lines tested are higher than their corresponding MICs. Tables S1 and S2 in the Supporting Materials show the Selectivity Index (SI). These SI have been calculated using the HC_50_/MIC values. The curves in Figure 6 and Figure 7 indicate that the IC_50_ values of some of these surfactants are higher than the HC_50_. However, we did not calculate the IC_50_ value because only five concentrations were tested. Therefore, it is expected that, for some of these compounds, using the IC_50_/MIC ratio would yield higher TI values. These results can be ascribed to their mode of action. It has been observed that the initial interaction of cationic surfactants with bacterial and fungal membranes occurs through physical perturbation of the membrane by electrostatic and hydrophobic interactions. The mechanism of action of the C_12_PAM mainly involves interactions with the fungal and bacterial membranes [21]. Differences in membrane charge and composition allow cationic surfactants to interact more strongly with fungal membranes than with mammalian membranes. The primary electrostatic interaction involves the positively charged cationic surfactants binding to negatively charged elements of fungal membranes, such as anionic phospholipids and acidic polysaccharides. The inner membrane of the *C. albicans* contains around 30% anionic components and is surrounded by a glucan-enriched hydrophobic cell wall [31]. In contrast, mammalian cell membranes are composed mainly of zwitterionic phospholipids, and any negatively charged phospholipids that do exist are generally confined to the inner leaflet, oriented toward the cytoplasm” [58]. Due to that, it was easier for these cationic surfactants to bind to the more negatively charged fungal membranes preferentially. Moreover, eukaryotic membranes also contain cholesterol, absent in the fungal membranes, that can prevent their disruption.

It is well established that cationic surfactants typically display greater cytotoxicity than their anionic and non-ionic counterparts [54]. Our findings show that while these cationic surfactants exhibit toxic effects at relatively low concentrations, such effects occur at levels higher than those commonly employed in biomedical and pharmacological applications. In this regard, it is noteworthy that the cytotoxicity of biologically active compounds can be reduced in physiological environments. For example, Crowston et al. observed that the factors present in human serum reduced the toxicity of mitomycin-C in fibroblasts [59], Dawgul et al. found that the cytotoxicity of a C_16_-lys-lys lipopeptide was reduced in physiological environments [36], and Lai et al. observed that the cytotoxicity of antimicrobial peptides was reduced by the plasma ingredients such as apolipoproteins and other lipoproteins [60].

### 3.5. Ecotoxicity

To evaluate the aquatic toxicity of the amino acid-derived surfactants, their aquatic toxicity was assessed through standardized assays using two model organisms: the freshwater microcrustacean *Daphnia magna* and the saltwater bacterium *Vibrio fischeri*. These organisms were selected as representative species for freshwater and marine environments, respectively, providing a comprehensive ecotoxicological assessment across different aquatic ecosystems.

The ecotoxicity of surfactants featuring dipeptide polar head groups (Phe-Arg and Trp-Arg) was investigated using *Daphnia magna* as the test organism (Table 5). The results demonstrated a clear inverse relationship between alkyl chain length and EC_50_ values, indicating that increased hydrophobicity of the surfactant molecule correlates with enhanced toxicity towards *Daphnia*. A significant increase in aquatic toxicity was observed with increasing alkyl chain length, a trend consistent with previous findings for other cationic surfactants [45,61,62].

Comparative analysis of the two dipeptide surfactant families (PANHCn and TANHCn) revealed distinct toxicity patterns. For shorter alkyl chain lengths (n ≤ 12), the Trp-Arg head group (TANHCn) exhibited higher toxicity toward *Daphnia* compared to Phe-Arg (PANHCn). This observation can be attributed to the greater inherent hydrophobicity of the tryptophan residue relative to phenylalanine. However, with longer alkyl chains (C14), the contribution of the hydrophobic tail becomes the dominant factor in toxicity, effectively diminishing the influence of the polar head group composition.

When comparing dipeptide-based surfactants with analogous surfactants possessing a single arginine polar head group (C12 alkyl chain), the latter demonstrated marginally lower toxicity toward *Daphnia*. This subtle difference may be attributed to the additional hydrophobic character contributed by the phenylalanine and tryptophan residues present within the dipeptide polar group.

When the aquatic toxicity of these amino acid-derived surfactants was benchmarked against conventional quaternary ammonium surfactants, such as benzalkonium chloride (BAC) and dodecyl dimethyl ammonium bromide (DDAB), the conventional surfactants displayed significantly higher toxicity towards *Daphnia*. The observed EC_50_ values for the quaternary ammonium compounds were one to two orders of magnitude lower (indicating higher toxicity) than those of the amino acid-based surfactants. Therefore, amino acid-derived surfactants are considerably less toxic than conventional quaternary ammonium surfactants, providing valuable information for developing more environmentally friendly alternatives.

The acute toxicity of amino acid-based surfactants bearing two amino acids and two cationic charges in the hydrophilic moiety was also evaluated against the marine bacterium *Vibrio fischeri*, a widely used organism in ecotoxicity testing due to its sensitivity and rapid response to toxic substances. Toxicity was quantified using the EC_50_ value, which represents the concentration of the compound causing a 50% inhibition in *Vibrio fischeri* bioluminescence after 15 min of exposure (Table 6). Lower EC_50_ values indicate higher toxicity. The results were analyzed with 95% confidence intervals to ensure statistical reliability.

The aquatic toxicity of surfactants with two amino acids in the polar group increased with increasing alkyl chain length, consistent with observations from *Daphnia* assays. The enhanced hydrophobicity of PANHC_n_ and TANHC_n_ surfactants due to alkyl chain elongation led to increased aquatic toxicity. Similarly, for surfactants with shorter alkyl chains (<C_12_), those containing Trp-Arg in the polar group showed somewhat higher toxicity than the Phe-Arg family, attributed to the greater hydrophobicity of the tryptophan residue compared to phenylalanine. However, this trend was not maintained for the TANHC_14_ surfactant, which showed lower toxicity (higher EC_50_ value) compared to C12. This deviation can be attributed to the reduced solubility of the C14 compound in the saline assay medium, which may have limited its bioavailability and apparent toxicity. The C_12_AM surfactant, containing a single amino acid and one charge in the polar group, presented aquatic toxicity similar to or slightly higher than the two-amino acid, two-charge families against luminescent bacteria.

Comparing the responses of the two microorganisms revealed distinct sensitivity patterns. *Vibrio fischeri* appears more uniformly sensitive to arginine-based surfactants across different chain lengths, with EC_50_ values showing less dramatic variation compared to *Daphnia*. This uniformity may be attributed to the saline medium used in bacterial assays, which could standardize the bioavailability and interaction of surfactants with bacterial cells. In contrast, *Daphnia magna* showed a much stronger dependence on alkyl chain length, with toxicity increasing dramatically as chain length increased from C10 to C14. The nature of the polar head group also played a more pronounced role in *Daphnia* toxicity, particularly for shorter chain surfactants, though this influence diminished with increasing alkyl chain length.

Hydrophobicity plays a key role in the aquatic toxicity of the amino acid-derived surfactants studied. It should be noted that the toxicity against all microorganisms and cells studied in this work, fungi, erythrocytes, 3T3 cells, HaCaT cells, *Daphnia magna,* and *Vibrio fischeri*, follows the same trend: it tends to increase with increasing the hydrophobicity values of the amino acid forming part of the surfactant’s polar group. Consequently, these biological properties can also be correlated with the cmc and the Rt; the toxicity against all tested microorganisms increases as the cmc decreases and the Rt increases. These findings demonstrate that molecular structure, particularly the composition of the polar head group and alkyl chain length, significantly influences the cytotoxic and ecotoxicological profile of amino acid-derived surfactants.

The differential responses observed between freshwater (*Daphnia magna*) and marine (*Vibrio fischeri*) test organisms highlight the importance of using multiple species in ecotoxicological assessments. The relationship between structural features and aquatic toxicity provides valuable insights for designing environmentally safer surfactants with reduced ecological impact across different aquatic ecosystems.

## 4. Conclusions

Despite extensive investigation into the antimicrobial efficacy of many biocides, including quaternary ammonium compounds (QACs), their cytotoxicity and ecotoxicity have received comparatively little attention. Furthermore, comprehensive experimental strategies concurrently evaluating antimicrobial effectiveness alongside toxicity toward mammalian cells and aquatic organisms are scarce. This study addresses this gap by presenting a systematic approach to designing small-molecule cationic surfactants with potent antimicrobial and antibiofilm properties, mimicking short antimicrobial peptides.

Synthesized in just three steps from renewable and inexpensive raw materials, these surfactants demonstrated robust activity against a broad spectrum of *Candida* strains, including the clinically challenging *C. auris*, and effectively eradicated fungal biofilms at low concentrations. Our findings consistently showed that hydrophobicity is a key physicochemical parameter influencing both antimicrobial efficacy and toxicity profiles.

While these compounds exhibited moderate cytotoxicity toward mammalian cells, their HC_50_ and IC_50_ values—measured against two different cell lines—were higher than their corresponding MIC values, suggesting a favorable therapeutic index. Critically, these amino acid-derived surfactants proved considerably less toxic to aquatic organisms like *Daphnia magna* and *Vibrio fischeri* than conventional QACs, with toxicity tending to increase with alkyl chain length.

This study highlights that chemical structure—especially the composition of the hydrophilic group and the length of the hydrophobic alkyl chain—plays a critical role in determining antifungal efficacy, cytotoxicity, and ecotoxicity, thereby providing key guidance for the development of environmentally safer antimicrobial agents.

Among all synthesized amphiphiles, C_12_PAM and C_12_TAM emerged as the most promising candidates. These compounds exhibited good biodegradability, effectively eradicated fungal biofilms at low concentrations, and importantly, presented HC_50_ and IC_50_ values that exceeded their MIC values against both bacteria and fungi.

The development of such biocompatible cationic surfactants holds significant promise. Their use could contribute to reducing reliance on traditional antibiotics in healthcare and agriculture, thereby helping to mitigate the rise of antimicrobial resistance. Moreover, given their favorable toxicity profiles, these compounds may also offer potential for other biomedical applications, such as gene therapy or drug delivery systems.

## Figures and Tables

**Table 1:** Surface tension parameters and Rt of the arginine-based amino acids.

	logP	cmc (mM)	cmc Range * (mM)	cmc Cond (mM) **	γ_c_ (mNm^−1^)	Rt (min)
C12AM	1.14	1.7	[1.1, 2.8]	6	29.5 ± 1.5	12.4
PNHC12	3.15	1.2	[0.7, 2.1]	1.2	26.6 ± 0.8	14.8
LPAM	2.03	0.7	[0.3, 1.8]	1.2	29.5 ± 2.7	15.4
LTAM	2.50	0.65	[0.3, 1.5]	0.5	32.1 ± 2.3	15.9
PANHC12	1.71	1.5	[0.7, 3.2]	2.2	30.4 ± 2.6	13.5
TANHC12	2.17	2.5	[1.0, 6.5]	0.6	28.7 ± 3.3	15.1
PANHC10	0.69	6.7	[1.9, 23]	7.2	30.6 ± 9.7	11.8
PANHC12	1.71	1.5	[0.7, 3.2]	2.2	30.4 ± 2.6	13.5
PANHC14	2.72	0.35	[0.13, 0.95]	0.7	28.7 ± 1.5	15.0
TANHC10	1.16	5.4	[3.8, 7.6]	1.7	30.6 ± 2.0	13.5
TANHC12	2.17	2.5	[1.0, 6.5]	0.6	28.7 ± 3.3	15.1
TANHC14	3.18	0.7	[0.4, 1.3]	0.3	32.8 ± 1.2	16.2

* Instead of incertitude intervals, a range is given because of the semilogarithmic nature of the surface tension plots. ** Conductivity values from reference [12].

**Table 2:** MIC values [μg/mL (μM)] of surfactants with C_12_ alkyl chains.

	BAC	C_12_AM	PNHC_12_	PANHC_12_	TANHC_12_	C_12_PAM	C_12_TAM
*C. albicans*	8 (24)	32 (78)	8 (22)	32 (56)	64 (108)	16 (29)	32 (54)
*C. tropicalis*	4 (12)	8 (19)	32 (88)	32 (56)	32 (54)	16 (29)	16 (27)
*C. parapsilosis*	4 (12)	16 (39)	32 (88)	32 (56)	32 (54)	16 (29)	16 (27)
*C. glabrata*	8 (24)	16 (39)	32 (88)	32 (56)	128 (216)	16 (29)	16 (27)
*C. rugosa*	8 (24)	64 (156)	32 (88)	64 (112)	4 (7)	4 (7)	8 (13)
*C. auris*	32 (98)	128 (312)	32 (88)	64 (112)	128 (216)	32 (58)	16 (27)
*C. jardinii*	16 (48)	64 (156)	32 (88)	32 (88)	64 (108)	64 (116)	32 (54)

**Table 3:** MIC values [μg/mL (μM)] of surfactants with two amino acids on the polar heads.

	PANHC_8_	PANHC_10_	PANHC_12_	PANHC_14_	TANHC_10_	TANHC_12_	TANHC_14_
*C. albicans*	>256 (>512)	128 (240)	32 (56)	16 (27)	64 (112)	64 (108)	16 (25)
*C. tropicalis*	256 (512)	128 (240)	32 (56)	8 (14)	64 (112)	32 (54)	4 (7)
*C. parapsilosis*	256 (512)	64 (120)	32 (56)	8 (14)	64 (112)	32 (54)	16 (25)
*C. glabrata*	256 (512)	64 (120)	32 (56)	8 (14)	64 (112)	64 (108)	8 (14)
*C. rugosa*	>256 (>512)	128 (240)	64 (112)	16 (27)	4 (7)	4 (7)	4 (7)
*C. auris*	>256 (>512)	256 (480)	64 (112)	16 (27)	128 (224)	128 (216)	16 (25)
*C. jardinii*	>256 (>512)	128 (240)	32 (56)	16 (27)	64 (112)	64 (108)	8 (14)

**Table 4:** HC_50_ values of the amino acid-based surfactants.

	HC_50_ μg/mL (μM)
C12AM	109 ± 0.6 (270 ± 1.7)
PNHC12	20 ± 0.6 (55 ± 1.7)
LPAM	23 ± 0.8 (42 ± 1.5)
LTAM	24 ± 0.8 (40 ± 1.6)
PANHC12	26 ± 1.2 (46 ± 2.2)
TANHC12	16 ± 0.3 (27 ± 0.6)
BAC	31 ± 0.3 (92 ± 0.8)
PANHC10	215 ± 22 (404 ± 42)
PANHC12	26 ± 1.2 (46 ± 2.2)
PANHC14	20 ± 0.5 (34 ± 0.8)
TANHC10	74 ± 2.8 (129 ± 5)
TANHC12	16 ± 0.3 (27 ± 0.6)
TANHC14	16 ± 0.18 (26 ± 0.3)

**Table 5:** Aquatic toxicity of amino acid-based surfactants and conventional quaternary ammonium surfactants against *Daphnia magna.* All values represent EC_50_ in μg/mL after 48 h of exposure. Values in parentheses indicate 95% confidence intervals.

Alkyl Chain Length (n)	PANHCn	TANHCn	C_12_AM	BAC ^a^	DDAB ^b^
10	22 (20–24)	9.7 (8.6–11)	-	-	0.15 (0.14–0.17)
12	3.4 (2.3–5.3)	3.0 (2.7–3.4)	4.4 (3.5–5.4)	0.039 (0.023–0.05)	-
14	0.71 (0.60–1.1)	0.85 (0.75–1.1)	-	-	-

Notes: ^a^ BAC is typically a mixture with alkyl chain lengths averaging between C12–C14 (70% C12 and 30% C14). ^b^ DDAB contains two C10 alkyl chains; therefore, it is listed in the C10 row.

**Table 6:** Acute toxicity of amino acid-based surfactants using the bacterium *Vibrio fischeri*. EC_50_ values in μg/mL (median effective concentration) and their 95% confidence intervals after 15 min of exposure.

Alkyl Chain Length (n)	PANHCn	TANHCn	C_12_AM
10	5.6 (5.3–5.9)	2.2 (1.8–2.8)	
12	3.0 (2.6–3.5)	1.9 (1.3–2.7)	1.9 (1.8–2.1)
14	1.8 (1.3–2.3)	2.6 (2.1–3.2)	

## Data Availability

The data supporting this study’s findings are available from the corresponding author upon reasonable request.

## Supplementary Materials

The following supporting information can be downloaded at: https://www.mdpi.com/article/10.3390/jox15050140/s1, Figure S1: MIC values (μg/mL) of surfactants with C12 alkyl chains; Table S1: Selectivity Index (HC50/MIC) of surfactants with C12 alkyl chains; Table S2: Selectivity Index (HC50/MIC) of surfactants with two amino acids on the polar heads.
