# Supplementary material for: Arginine-Derived Cationic Surfactants Containing Phenylalanine and Tryptophan: Evaluation of Antifungal Activity, Biofilm Eradication, Cytotoxicity, and Ecotoxicity
Source: J Xenobiot. 2025 Sep 3;15(5):140. doi: 10.3390/jox15050140 (PMC12452389; doi:10.3390/jox15050140)
Supplement: Supplementary file 1 [file jox-15-00140-s001.zip › jox-3709608-supplementary.pdf]

Arginine-Derived Cationic Surfactants Containing Phenylalanine and Tryptophan: Evaluation of Antifungal Activity, Biofilm Eradication, Cytotoxicity, and Ecotoxicity

M.T. García [1], M.C. Morán [2,3], R. Pons [1], Z. Hafidi [1], E. Bautista [1], S. Vazquez [1,4] and L. Pérez [1*]

[1]Department of Surfactants and Nanobiotechnology, Institute for Advanced Chemistry of Catalonia (IQAC-CSIC), C/ Jordi Girona 18-26, 08034, Barcelona, Spain

[2] Secció de Fisiologia, Departament de Bioquímica i Fisiologia, Facultat de Farmàcia i Ciències de l'Alimentació, Universitat de Barcelona, Avda. Joan XXIII 27-31, 08028 Barcelona, Spain;

[3]Institut de Nanociència i Nanotecnologia—IN2UB, Universitat de Barcelona, Avda. Diagonal, 645, 08028 Barcelona, Spain

[4]Doctoral Program in Biotechnology, Facultat de Farmàcia i Ciències de l'Alimentació, Universitat de Barcelona, Avda. Diagonal 643, 08028 Barcelona, Spain.

* Correspondence: Lourdes.perez@iqac.csic.es

Contents

Figure S1: MIC values (μg/mL) of surfactants with C12 alkyl chains.

Table S1: Selectivity Index (HC50/MIC) of surfactants with C12 alkyl chains

Table S2: . Selectivity Index (HC50/MIC) of surfactants with two amino acids on the polar heads.

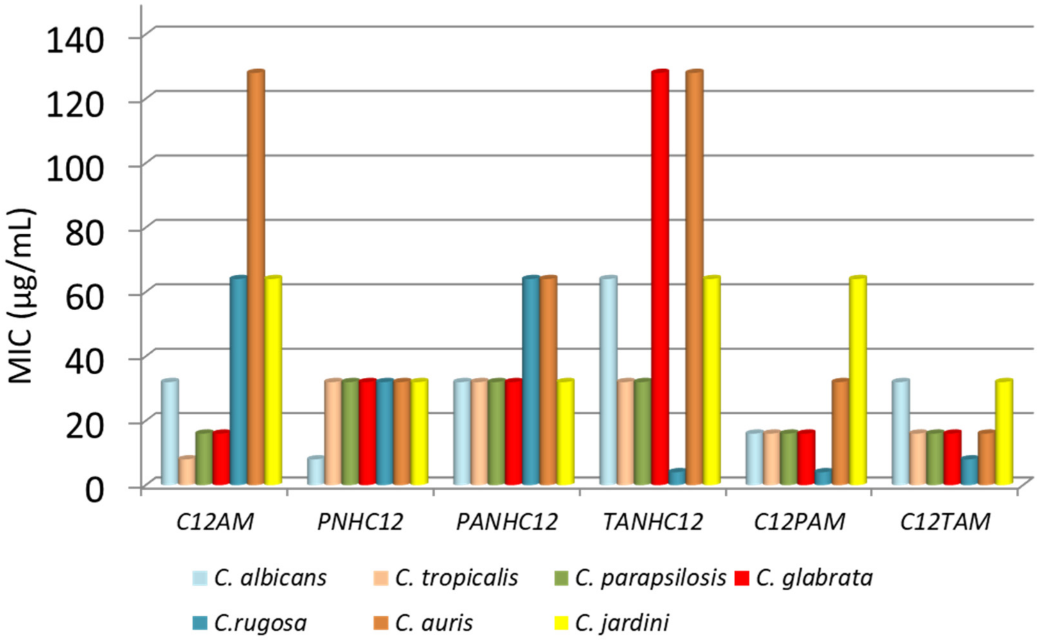

Figure S1. MIC values (µg/mL) of surfactants with C_{12} alkyl chains.

Table S1. Selectivity Index (HC_{50}/MIC) of surfactants with C_{12} alkyl chains

	BAC	C_{12}AM	PNHC_{12}	PANHC_{12}	TANHC_{12}	C_{12}PAM	C_{12}TAM
C. albicans	3.8	3.4	2.5	0.8	0.25	1.4	0.7
C. tropicalis	7.6	14	0.6	0.8	0.5	1.4	1.4
C. parapsilosis	.7.6	7	0.6	0.8	0.5	1.4	1.4
C. glabrata	3.8	7	0.6	0.8	0.12	1.4	1.4
C. rugosa	3.8	1.7	0.6	0.4	3.8	6	3
C. auris	0.9	0.9	0.6	0.4	0.12	0.7	1.4
C. jardini	1.9	1.7	0.6	0.8	0.25	0.4	0.7

Table S2. Selectivity Index (HC_{50}/MIC) of surfactants with two amino acids on the polar heads.

	PANHC_{10}	PANHC_{12}	PANHC_{14}	TANHC_{10}	TANHC_{12}	TANHC_{14}
C. albicans	1.6	0.8	1.2	1.15	0.25	1.1
C. tropicalis	1.6	0.8	2.4	1.15	0.5	3.7
C. parapsilosis	3.7	0.8	2.4	1.15	0.5	1.1
C. glabrata	3.7	0.8	2.4	1.15	0.25	1.9
C. rugosa	1.6	0.4	1.2	18	4	3.7
C. auris	0.8	0.4	1.2	0.6	0.1	1.1
C. jardini	1.6	0.8	1.2	1.15	0.25	1.9
